# Cognitive impairment negatively impacts allied health service uptake: Investigating the association between health and service use

**DOI:** 10.1016/j.ssmph.2020.100720

**Published:** 2020-12-13

**Authors:** Catherine A. MacLeod, Feifei Bu, Alasdair C. Rutherford, Judith Phillips, Robert Woods, in collaboration with the CFAS WALES research team

**Affiliations:** a Dementia Services Development Centre Wales, School of Health Sciences, Bangor University, UK; b Department of Behavioural Science and Health, University College London, UK; c Sociology, Social Policy & Criminology, University of Stirling, UK; d Dementia and Ageing, University of Stirling, UK

**Keywords:** Health service use, Social care, Allied health, Older adults, Dementia, Cognitive impairment

## Abstract

There is widespread concern about the potential impact on health and social care services of the ageing population and long-term health conditions, such as dementia. To effectively plan services it is important to understand current need and use and identify gaps in provision. Using data from the Cognitive Function and Ageing Study Wales (CFAS Wales), we used logistic regression to model the relationship between health (self-rated health, cognitive impairment, and activities of daily living), and the use of health and care services. CFAS Wales is a longitudinal cohort study of people aged 65 years and over, in two areas in Wales, UK, over-sampling those aged 75 years and over. Participants (n = 3593) answered a wide range of health and lifestyle questions and completed a variety of cognitive and physical health assessments. Data from 3153 people from wave 1 and 1968 people from wave 2 were analysed.

As anticipated we found poorer health, on some indicators, predicted greater service use, including social care, hospital, general practitioner, and nursing services. However, cognitive impairment did not predict greater service use, except for social care. Controlling for age, sex, socio-economic status, social connection indices and area environment, conversely we found lower reported uptake of allied health services by people with cognitive impairment. Further analysis showed that people with a cognitive impairment were less likely to report having a sight-check or seeing a dentist in the previous year, a finding replicated in wave 2. These differences were not explained by transportation issues. In contrast, we did not find a significant difference in reported uptake of hearing checks or physiotherapist use, with mixed evidence of differences in chiropodist visits. Not accessing these preventative services may not only exacerbate existing conditions but have further downstream negative consequences for health and well-being in people who are cognitively impaired.

## Highlights

• Medical and social care service use typically increased as health decreased.
• Allied health services uptake decreased for those with a cognitive impairment.
• Cognitive impairment significantly lowered odds of sight checks and dentist visits.
• Inequalities in sight and dental checks remain after factoring in accessibility.
• Routine health checks remain important even if facing other health challenges.

## 1 Introduction

Across the world populations are ageing, with increases in both the absolute number and proportion of older people making up populations (World Health Organisation, 2015). Whilst people are living longer than ever before, UK data shows that changes in healthy life expectancy are smaller than changes in life expectancy, suggesting that years living in poor health increased more than years in good health (Office for National Statistics, 2019). A major factor is the increased number of people living with dementia - estimated to rise in the UK from 850,000 in 2015 to over 1 million by 2025 (Prince et al., 2014). A core concern for service providers and commissioners is the impact of this ageing population on demand for services and the sustainability of existing services (Licchetta & Stelmach, 2016).

The Andersen behavioural model of health service use (Andersen, 1995) identifies three characteristics that influence a person's access to and use of health services: predisposing factors (including individual socio-cultural characteristics such as social structures, health beliefs, and demographics), enabling factors (reflecting the logistics of getting care including the means to access services, e.g. finance or transportation, and availability of services in the community, e.g. waiting lists), and need factors (relating to perceived need for help, for example how people experience their symptoms and the importance they give them; and evaluated need, reflecting professional decisions about an individual's need). Whilst the Andersen model focuses on health services, this could also be applied to social care and other forms of assistance.

Considering a broader range of assistance that might come from both personal and professional providers, Canvin, MacLeod, Windle, and Sacker (2018) identified a four-stage recursive process that people engaged in when evaluating their need for assistance: acknowledgement of decline; perceived impact on usual activities and independence; preparedness to receive assistance; and opportunity to assert need. Canvin et al. (2018) found that older adults did not always seek help or delayed accessing services even when needed, engaging in self-management, with unsolicited or emergency interventions by third parties leading to assistance or service use.

There is a perception that as we get older our health is more likely to decline, our demand for services will increase and we become more dependent (World Health Organisation, 2015). However, “chronological age is only loosely associated with levels of functioning”, with the older population having a diverse range of capabilities and health needs (World Health Organisation, 2015, p. 16, p.16). Whilst health needs are an important factor in service use (Andersen, 1995), actual need does not always equal demand for or uptake of services (Canvin et al., 2018; Walters, Iliffe, & Orrell, 2001). The inverse care law (Hart, 1971), suggests those in most need of health care are least likely to receive it, whereas those with least need often use health services more and more effectively. The modification of expectations for health and well-being as we age (Sarkisian, Hays, & Mangione, 2002), along with a normalisation of ill-health symptoms, particularly amongst deprived groups (Dixon-Woods et al., 2005) can negatively affect health service uptake.

Health and social care services have a variety of roles, including prevention, healing, and improving well-being and quality of life. In order to effectively plan services, it is important to look beyond chronological age, to look at service use in relation to health (including cognitive impairment) as an indicator of need, to understand how services are currently being used, and identify where there may be gaps in service uptake.

There have been long-standing concerns regarding access to and up-take of health and social care services by people with cognitive impairment (e.g. Røsvik et al., 2020). The current study offers the opportunity to examine whether there have been improvements over time and whether the influence of national dementia plans and dementia guidelines have made a difference to service use. We use a similar methodology and have overlapping geographical coverage with a study by Burholt, Wenger, and Scott (1997) who examined contact with formal health and social care services in areas of England and Wales. They found that a higher percentage of people living with dementia accessed these services than those not living with dementia, although with a relatively small sample size many of these differences failed to reach statistical significance. Only two services showed significant differences across both urban and rural populations, with a higher percentage of people living with dementia visiting a geriatrician, and a lower percentage of people living with dementia visiting a dentist compared to those not living with dementia. This paper examines the association between use of a variety of health and social care services and a broader range of need variables, including self-rated health, cognitive health, and activities of daily living in a large sample of adults aged 65 years and over, allowing adjustment for demographic, social, and environmental factors. Whilst we expect service use to be, in general, driven by need, we will explore whether there is an interaction between particular need indicators and particular types of service.

## 2 Methods

### 2.1 Study design and population

Data comes from the Cognitive Function and Ageing Study Wales (CFAS Wales), a longitudinal cohort study investigating health and cognitive function of people aged 65 years and over living in areas of North (Gwynedd and Ynys Môn) and South (Neath, Port Talbot) Wales, UK (Woods et al., 2019). Participants were randomly sampled from primary care lists from 2011 to 2013, stratified by age group (65–74/75+ years) to ensure equal representation.

The North Wales Research Ethics Committee (West) granted ethical approval for the study (reference 10/NNo01/37), and participants gave written consent to take part. Computer-assisted personal interviews were conducted in either English or Welsh, depending on participant preference, and participants were invited to take part in a follow-up interview two years later. In wave 1, 3593 participants were interviewed, with 2236 participants completing the wave 2 interview. Table 1 provides a summary of participant characteristics at baseline. From the full sample, 99.2% of participants identified themselves as white, matching the ethnic makeup of the over 65's in Wales (99.2%) between 2013 and 2015 (Welsh Government, 2019). Due to the small number of non-white participants (<1%) ethnicity is not included in analyses. CFAS Wales is a sister project to CFAS II in England following the same sampling structure and basic study design. CFAS Wales included additional questions on topics including language, social networks, resilience, and nutrition. For further details about sampling, study design, and response rates see Matthews et al. (2013) and Clare et al. (2017).

**Table 1:** Participant numbers with sample percentages and means with standard deviations (SD) by health and covariate variables.

	Full Sample (n = 3593)	Analysis Sample (n = 3153)
	N (%) or Mean (SD)	N (%) or Mean (SD)
**Self-Rated Health**		
Excellent	740 (21%)	684 (22%)
Good	1574 (44%)	1468 (47%)
Fair	850 (24%)	776 (25%)
Poor	249 (7%)	225 (7%)
*Missing*	*180 (5%)*	*0 (0%)*
**Cognitive Function**		
No Impairment	2497 (70%)	2377 (75%)
Impairment	983 (27%)	776 (25%)
*Missing*	*113 (3%)*	*0 (0%)*
**Activities of Daily Living**		
No disability	2162 (60%)	2031 (64%)
IADL disability	786 (22%)	721 (23%)
ADL-IADL disability	570 (16%)	401 (13%)
*Missing*	*75 (2%)*	*0 (0%)*
**Age**		
Mean in years (SD; range)	75 (7; 65–102)	74 (7; 65–102)
*Missing*	*0 (0%)*	*0 (0%)*
**Sex**		
Male	1619 (45%)	1486 (47%)
Female	1974 (55%)	1667 (53%)
*Missing*	*0 (0%)*	*0 (0%)*
**Socio-economic Status**		
Higher managerial, administrative & professional	261 (7%)	248 (8%)
Lower managerial, administrative & professional	758 (21%)	725 (23%)
Intermediate occupations	509 (14%)	486 (15%)
Small employers & own account workers	397 (11%)	373 (12%)
Lower supervisory & technical occupations	409 (11%)	387 (12%)
Semi-routine occupations	500 (14%)	459 (15%)
Routine occupations	485 (14%)	435 (14%)
Never worked & long-term unemployed	41 (1%)	40 (1%)
*Missing*	*233 (6%)*	*0 (0%)*
**Marital Status**		
Married/Cohabiting	2185 (61%)	2005 (64%)
Single	155 (4%)	130 (4%)
Widowed	997 (28%)	799 (25%)
Divorced/Separated	250 (7%)	219 (7%)
*Missing*	*6 (* ≤ *1%)*	*0 (0%)*
**Accommodation Type**		
Without Assistance	3414 (95%)	3077 (98%)
With Assistance	176 (5%)	*76 (2%)*
*Missing*	*3 (* ≤ *1%)*	*0 (0%)*
**Household Composition**		
Alone	1183 (33%)	1036 (33%)
With Other	2310 (64%)	2117 (67%)
*Missing*	*100 (3%)*	*0 (0%)*
**Social Engagement**		
Mean (SD; range)	15 (6; 0–30)	15 (6; 0–30)
*Missing*	*46 (1%)*	*0 (0%)*
**Deprivation**		
High Deprivation	1194 (33%)	1018 (32%)
Medium Deprivation	1188 (33%)	1048 (33%)
Low Deprivation	1211 (34%)	1087 (34%)
*Missing*	*0 (0%)*	*0 (0%)*
**Rurality**		
Urban	1464 (41%)	1267 (40%)
Rural	2129 (59%)	1886 (60%)
*Missing*	*0 (0%)*	*0 (0%)*
**Centre**		
North Wales	1830 (51%)	1628 (52%)
South Wales	1763 (49%)	1525 (48%)
*Missing*	*0 (0%)*	*0 (0%)*

### 2.2 Service use measures

Service use was measured by respondent self-report using a binary yes/no response. Services were divided into five main categories: social care (in the four weeks prior to interview used home help, meals on wheels, occupational therapist, social worker, or day centre, or received respite care in the last year); hospital (in the four weeks prior to interview visited a day hospital, in the three complete calendar months before interview visited the casualty/emergency or outpatient department of a hospital, or in the year before interview been in hospital for treatment as a day patient or inpatient); allied health (in the four weeks prior to interview visited a chiropodist, physiotherapist, or speech therapist, or in the year before interview had eyesight tested by an optician, had a hearing test, or seen the dentist), general practitioner (GP - visited the GP/doctor in the four weeks prior to interview), nursing (used any nursing services in the 4 weeks prior to interview). Participants were considered a service user if they used at least one service in the category. Service categories were only classified as missing data if there were no responses for all services making up that category. Table 2 shows number and percentage of service users/non-users for each category. Each service use category was missing 3% of full sample data.

**Table 2:** Participant numbers with sample percentages by service use categories.

	Analysis Sample (n = 3153)
	N (%)
**Social Care**	
Service Non-Users	3008 (95%)
Service Users	145 (5%)
**Hospital**	
Service Non-Users	1940 (62%)
Service Users	1213 (38%)
**Allied Health Services**	
Service Non-Users	315 (10%)
Service Users	2838 (90%)
**GP**	
Service Non-Users	1986 (63%)
Service Users	1167 (37%)
**Any Nursing Services**	
Service Non-Users	2769 (88%)
Service Users	384 (12%)

### 2.3 Health measures

Health measures include self-rated health (SRH), cognitive function, and activities of daily living (ADL). SRH was measured by asking participants to indicate if their own health in general was excellent, good, fair, or poor for someone of their age. Cognitive function was dichotomised into no impairment and impairment groups, with cognitive impairment defined as having either a Mini-Mental State Examination score (Folstein, Folstein, & McHugh, 1975) of less than 26 or a study dementia diagnosis indicated by either the Geriatric Mental State - Automated Geriatric Examination for Computer-Assisted Taxonomy (Copeland, Dewey, & Saunders, 1991) algorithm or as assessed by a medically qualified member of the study team through review of participant and informant interviews, and interviewer vignettes (Matthews et al., 2013). Activities of daily living (Katz, 1983; Lawton & Brody, 1969) was classified into four groups (see Cognitive Function & Ageing Study, 2015(CFA, 2015)) (1) those with no activities of daily living (ADL) or instrumental activities of daily living (IADL) disability, (2) those with IADL disability only, (3) those with ADL and IADL disabilities, and (4) unclassified or missing. ADLs reflect basic care functions, including washing and dressing, whereas IADLs reflect activities needed to live independently, such as housework and shopping.

### 2.4 Covariates and modifiers

Covariates and modifier variables were divided into three groups: demographics, social connection, and environment. Demographic variables included age, sex, and National Statistics Socio-economic classification (NS-SEC8). NS-SEC8 classification includes higher managerial, administrative & professional; lower managerial, administrative & professional; intermediate occupations; small employers and own account workers; lower supervisory and technical occupations; semi-routine occupations; routine occupations; never worked and long-term unemployed (Office for National Statistics, 2016).

Social connection variables include marital status (married/cohabitating, single, widowed, or divorced/separated); accommodation type, categorised as either living in accommodation with assistance (defined as living in a granny flat, warden controlled flat, care home, or long stay hospital) or without assistance; household composition (living alone, or living with others); and social engagement, measured by the Lubben Social Network Scale – 6 (Lubben, 1988; Lubben et al., 2006), a scale of 0–30 with lower scores indicating higher levels of isolation.

Environment variables include area level measures of deprivation, as indicated by the Welsh Index of Multiple Deprivation (WIMD) 2014 (Welsh Government, 2015), rurality (urban or rural) as indicated by the 2011 Rural Urban Classification (Bibby & Brindley, 2013), and centre (North or South Wales). The WIMD is a composite measure including income, employment, health, education, access to services, community safety, physical environment, and housing indicators. Areas in Wales are ranked with lower ranks representing a more deprived area. For this study, WIMD ranks were divided into three equal sized groups, indicating if a participant lived in a high, medium, or low deprivation area. Data from wave 2 additionally included a variable on service accessibility using usual forms of transport including access to the optician, dentist, and chiropodist. Service access was grouped into two levels, no difficulty (very/quite easy/no wish to go), and difficulty (quite/very difficult/unable to go).

### 2.5 Data analysis

Using data from wave 1, the effect of health on service use was assessed using logistic regression. Four models were estimated for each category of health and social care service (social care, hospital use, allied health services, GP, and nursing services): model 1 (base model) included three measures of health (SRH, cognitive function, ADL); model 2 adjusted for age, sex, and socio-economic status; model 3 further adjusted for social connection factors, including marital status, accommodation type, household composition, and social engagement; model 4 further adjusted for environment factors, including area deprivation, rurality, and centre. Participants (12%) were excluded if they were missing data on any variables used in the analysis, giving a final analysis sample of 3153 participants. Table 1 provides a summary of participant characteristics for the analysis sample.

The majority of participants used at least one allied health service, contrasting the opposite pattern of usage for the other four types of service examined. Furthermore, there was evidence that cognitive impairment was associated with lower uptake of allied health services. To explore these data patterns in more detail follow-up analysis used logistic regression to assess the effect of cognitive function on individual allied health services (sight test, hearing test, dentist, chiropodist, physiotherapist) in waves 1 and 2, using the same model structure outlined above. Speech therapy was excluded due to the small number of service users (n < 5). Participants were again excluded from analysis if missing data on any included variables (with service variables changing in the follow-up analysis from categories to individual services), giving a follow-up analysis sample of 3152 for wave 1, and 1968 for wave 2. Additional wave 2 analyses added a fifth model, adjusting for service access, to assess the effect of cognitive function on use of sight tests (n = 1955), dentist visits (n = 1958), and chiropodist visits (n = 1943).

All analyses were conducted using Stata V14.2. Average marginal effects were computed using the SPost mchange command (Long & Freese, 2014). CFAS Wales data version 3.0 was used in the analysis.

## 3 Results

Only 5% of participants were classified as users of social care, 38% used hospital, 37% used GP and 12% used nursing services; however, 90% of people used at least one allied health service (Table 2). Logistic regression results for each service by SRH, cognitive function, and ADL are presented in Table 3. Average marginal effects of service use can be found in Supplementary Table S1.

**Table 3:** Logistic regression odds ratios and 95% CIs for service use regressed on health (n = 3153).

	Model 1	Model 2	Model 3	Model 4
SOCIAL CARE				
Self-Rated Health				
Excellent	Reference	Reference	Reference	Reference
Good	0.72 (0.41–1.26)	0.78 (0.44–1.37)	0.72 (0.41–1.29)	0.74 (0.41–1.32)
Fair	0.71 (0.40–1.26)	0.88 (0.48–1.57)	0.78 (0.43–1.43)	0.81 (0.44–1.50)
Poor	0.40* (0.20–0.82)	0.68 (0.32–1.45)	0.62 (0.28–1.33)	0.66 (0.30–1.43)
**Cognitive Function**				
No Impairment	Reference	Reference	Reference	Reference
Impairment	2.51*** (1.75–3.61)	2.06*** (1.40–3.03)	1.88** (1.26–2.80)	1.90** (1.28–2.83)
**Activities of Daily Living**				
No disability	Reference	Reference	Reference	Reference
IADL disability	7.06*** (4.05–12.31)	4.97*** (2.78–8.89)	4.76*** (2.65–8.55)	4.83*** (2.69–8.67)
ADL-IADL disability	22.21*** (12.79–38.57)	14.35*** (7.93–25.96)	14.63*** (8.03–26.65)	14.85*** (8.15–27.04)
**HOSPITAL**				
**Self-Rated Health**				
Excellent	Reference	Reference	Reference	Reference
Good	1.42** (1.16–1.75)	1.44*** (1.17–1.77)	1.44*** (1.18–1.77)	1.44** (1.17–1.77)
Fair	2.12*** (1.68–2.68)	2.15*** (1.70–2.72)	2.16*** (1.70–2.74)	2.17*** (1.71–2.75)
Poor	2.71*** (1.92–3.81)	2.80*** (1.97–3.99)	2.82*** (1.98–4.02)	2.87*** (2.02–4.10)
**Cognitive Function**				
No Impairment	Reference	Reference	Reference	Reference
Impairment	0.95 (0.79–1.13)	0.92 (0.76–1.11)	0.93 (0.77–1.12)	0.94 (0.78–1.14)
**Activities of Daily Living**				
No disability	Reference	Reference	Reference	Reference
IADL disability	1.79*** (1.49–2.16)	1.81*** (1.48–2.21)	1.80*** (1.48–2.20)	1.84*** (1.51–2.25)
ADL-IADL disability	2.14*** (1.68–2.73)	2.04*** (1.58–2.64)	2.07*** (1.59–2.68)	2.11*** (1.63–2.74)
**ALLIED HEALTH**				
**Self-Rated Health**				
Excellent	Reference	Reference	Reference	Reference
Good	1.13 (0.84–1.53)	1.14 (0.85–1.55)	1.17 (0.86–1.59)	1.17 (0.86–1.59)
Fair	1.40 (0.96–2.04)	1.45 (1.00–2.12)	1.53* (1.04–2.23)	1.54* (1.05–2.25)
Poor	0.78 (0.48–1.27)	0.79 (0.48–1.31)	0.86 (0.52–1.44)	0.88 (0.53–1.47)
**Cognitive Function**				
No Impairment	Reference	Reference	Reference	Reference
Impairment	0.49*** (0.38–0.64)	0.54*** (0.42–0.71)	0.60*** (0.45–0.78)	0.61*** (0.46–0.80)
**Activities of Daily Living**				
No disability	Reference	Reference	Reference	Reference
IADL disability	1.12 (0.82–1.54)	1.16 (0.83–1.61)	1.20 (0.86–1.68)	1.22 (0.87–1.71)
ADL-IADL disability	0.95 (0.65–1.39)	1.00 (0.67–1.49)	1.05 (0.70–1.57)	1.09 (0.72–1.64)
**GENERAL PRACTITIONER (DOCTOR)**				
**Self-Rated Health**				
Excellent	Reference	Reference	Reference	Reference
Good	1.45*** (1.18–1.77)	1.46*** (1.19–1.79)	1.47*** (1.20–1.81)	1.47*** (1.20–1.81)
Fair	2.14*** (1.69–2.71)	2.19*** (1.73–2.77)	2.22*** (1.75–2.82)	2.24*** (1.76–2.84)
Poor	3.08*** (2.19–4.33)	3.28*** (2.31–4.65)	3.36*** (2.37–4.78)	3.42*** (2.40–4.87)
**Cognitive Function**				
No Impairment	Reference	Reference	Reference	Reference
Impairment	0.87 (0.73–1.04)	0.87 (0.72–1.04)	0.88 (0.73–1.06)	0.89 (0.74–1.07)
**Activities of Daily Living**				
No disability	Reference	Reference	Reference	Reference
IADL disability	1.29** (1.07–1.56)	1.27* (1.04–1.55)	1.27* (1.04–1.56)	1.29* (1.06–1.58)
ADL-IADL disability	1.34* (1.05–1.72)	1.28 (0.99–1.66)	1.29 (0.99–1.67)	1.31* (1.01–1.71)
**NURSING SERVICES**				
**Self-Rated Health**				
Excellent	Reference	Reference	Reference	Reference
Good	1.13 (0.82–1.54)	1.14 (0.83–1.56)	1.18 (0.86–1.62)	1.16 (0.84–1.60)
Fair	1.53* (1.08–2.16)	1.54* (1.09–2.19)	1.63** (1.14–2.32)	1.68** (1.17–2.41)
Poor	1.90** (1.21–2.99)	1.85* (1.16–2.94)	2.06** (1.28–3.30)	2.38*** (1.47–3.86)
**Cognitive Function**				
No Impairment	Reference	Reference	Reference	Reference
Impairment	0.97 (0.75–1.24)	1.03 (0.79–1.34)	1.09 (0.83–1.42)	1.17 (0.89–1.55)
**Activities of Daily Living**				
No disability	Reference	Reference	Reference	Reference
IADL disability	1.32 (1.00–1.74)	1.40* (1.05–1.88)	1.42* (1.05–1.90)	1.59** (1.17–2.15)
ADL-IADL disability	2.17*** (1.57–2.99)	2.33*** (1.65–3.28)	2.40*** (1.70–3.39)	2.74*** (1.93–3.90)

Odds Ratios (95% CI). *p < 0.05, **p < 0.01, ***p < 0.001.

M1 = Self-Rated Health, Cognition, Activities of Daily Living.

M2 = M1 + Age, Sex, Socio-economic Status.

M3 = M2 + Marital Status, Accommodation Type, Household Composition, Social Engagement.

M4 = M3 + Area Deprivation, Rurality, Centre.

### 3.1 Social care service use

The base model shows a significant association between poor SRH and social care use, with odds of using a social care service decreasing by a factor of 0.40 for those in poor health compared to odds of someone in excellent health (p = 0.013). However, this association did not reach significance when adjusting for covariates and modifiers in subsequent models. Across all models, use of social care services was significantly associated with having a cognitive impairment, and having either an IADL or ADL-IADL disability. In model 4, participants with a cognitive impairment had odds of using a social care service 1.90 times larger than odds for those without cognitive impairment (p = 0.002). Having an IADL disability increases the odds of using a social care service 4.83 times (p < 0.001) and having an ADL/IADL disability 14.85 times (p < 0.001). The probability of using social care services increased with increasing levels of activities of daily living disability (p < 0.001).

### 3.2 Hospital service use

Across all models, hospital service use is not associated with cognitive impairment, but is significantly associated with SRH, and with having either an IADL or ADL-IADL disability. In model 4, odds of using a hospital service are 1.44 times larger for those reporting good health (p = 0.001), 2.17 times larger for those reporting fair health (p < 0.001), and 2.87 times larger for those reporting poor health (p < 0.001), compared to those reporting their health to be excellent. There are also significant increases in the probability of using hospital services for those rating their health as fair (p < 0.001) or poor (p < 0.001) compared to those rating their health as good. There is no significant change in probability of use for those with poor compared to fair SRH. The odds of using a hospital service are 1.84 times greater if the person has an IADL disability (p < 0.001), and 2.11 times greater for someone with an ADL/IADL disability, compared with no disability (p < 0.001).

### 3.3 Allied health service use

There are no significant associations between SRH and allied health service use in the base and first models, however adjusting for social connection variables in model 3 resulted in a significant association in models 3 and 4 with fair SRH. In model 4, odds of using an allied health service are 1.54 times larger for those reporting fair health compared to excellent health (p = 0.026). In contrast, the probability of using an allied health service, is significantly decreased for those with poor compared to those with fair SRH (p = 0.041). Across all models there is a significant association between cognition and allied health service use. In model 4, odds of a participant with cognitive impairment using an allied health service are 0.61 times smaller than odds for those without a cognitive impairment (p < 0.001). There are no significant associations between activities of daily living and allied health service use.

### 3.4 General practitioner service use

Across all models there is a significant association between GP use and SRH, odds of visiting the GP increased 1.47 times for good SRH (p < 0.001), 2.24 times for fair SRH (p < 0.001), and 3.42 times for poor SRH (p < 0.001), compared to excellent SRH in model 4. The estimated probabilities indicate a significant increase in GP use as SRH worsens (fair – good health p < 0.001; poor – good health p < 0.001; poor – fair health p = 0.008). There is no significant association between cognitive function and GP use, but significant associations between activities of daily living and GP use. In model 4, odds of visiting the GP are 1.29 times higher for those with an IADL disability (p = 0.012), and 1.31 times for those with an ADL-IADL disability, compared to those with no disability (p = 0.041). The probability of GP use does not differ significantly between those with IADL and ADL-IADL disabilities.

### 3.5 Nursing service use

There is a significant association between nursing service use and fair and poor SRH across all models. In model 4, odds of using nursing services are 1.68 (p = 0.005) and 2.38 (p < 0.001) times larger for fair and poor SRH respectively, compared to excellent SRH. There is no significant difference in odds between good and excellent SRH, but the probability of nursing service use is greater for fair (p = 0.012) and poor SRH (p = 0.004) compared to good SRH. The probability of nursing service use does not differ significantly between fair and poor SRH. There is no significant association between cognitive function and nursing service use. There are significant associations between activities of daily living and nursing service use, with significant associations across models 1–3 for IADL disability and all four models for ADL-IADL disability compared to no impairment. In model 4, odds of using a nursing service are 1.59 times higher for those with an IADL disability (p = 0.003), and 2.74 times higher for those with an ADL-IADL disability (p < 0.001), compared to those with no disability. The probability of using nursing services was significantly greater for those with ADL-IADL disability, compared to those with IADL disability only (p = 0.003).

### 3.6 Allied health breakdown by cognition

Across most service categories the overall pattern of change is as expected, with poorer health resulting in larger odds and increased probability of using health and social care services. The major exception to this pattern is the smaller odds and decrease in probability of using an allied health service associated with cognitive impairment. To explore this further, follow-up analyses looking at the relationship between cognitive impairment and each allied health service were conducted.

The upper half of Table 4 shows the wave 1 logistic regression results for each individual allied health service by cognitive function. Average marginal effects of using each service by cognitive function in wave 1 can be found in Supplementary Table S2. No association between cognitive impairment and hearing checks or physiotherapy use were identified. Across all models there is a significant association between cognitive impairment and having a sight test in the year before interview, and between cognitive impairment and visiting the dentist in the year before interview. In model 4, odds of a participant with cognitive impairment having their sight tested are 0.77 times smaller than odds for those without a cognitive impairment (p = 0.008), and odds of visiting a dentist 0.76 times smaller (p = 0.005). The base model showed a significant association between cognitive impairment and chiropody use, with a person with a cognitive impairment having odds 1.33 times larger than those without a cognitive impairment, however this was attenuated when adjusting for age, sex, and socio-economic status in model 2, and did not reach significance in subsequent models.

**Table 4:** Logistic regression odds ratios and 95% CIs for each allied health service use regressed on cognitive function in wave 1 (n = 3152) and wave 2 (n = 1968).

		Model 1	Model 2	Model 3	Model 4
**Wave 1**	**SIGHT TEST** (users n = 2168, 69%)	0.81* (0.68–0.97)	0.77** (0.63–0.92)	0.78* (0.64–0.94)	0.77** (0.64–0.93)
	**HEARING TEST** (users n = 457, 15%)	1.07 (0.85–1.35)	0.93 (0.73–1.19)	0.95 (0.74–1.21)	0.95 (0.74–1.22)
	**DENTIST** (users n = 2083, 66%)	0.53*** (0.44–0.62)	0.69*** (0.58–0.84)	0.74** (0.61–0.89)	0.76** (0.63–0.92)
	**CHIROPODIST** (users n = 509, 16%)	1.33* (1.07–1.66)	1.04 (0.82–1.32)	1.07 (0.84–1.36)	1.06 (0.84–1.35)
	**PHYSIOTHERAPIST** (users n = 134, 4%)	0.71 (0.46–1.10)	0.92 (0.58–1.46)	0.98 (0.62–1.55)	1.05 (0.66–1.66)
**Wave 2**	**SIGHT TEST** (users n = 1403, 71%)	0.77* (0.61–0.98)	0.73* (0.57–0.93)	0.73* (0.57–0.94)	0.73* (0.57–0.94)
	**HEARING TEST** (users n = 364, 19%)	1.39* (1.06–1.82)	1.28 (0.97–1.71)	1.26 (0.94–1.68)	1.25 (0.94–1.67)
	**DENTIST** (users n = 1397, 71%)	0.46*** (0.36–0.58)	0.55*** (0.43–0.70)	0.57*** (0.44–0.73)	0.58*** (0.45–0.74)
	**CHIROPODIST** (users n = 278, 14%)	0.82 (0.59–1.13)	0.66* (0.47–0.93)	0.64* (0.46–0.90)	0.63** (0.45–0.89)
	**PHYSIOTHERAPIST** (users n = 89, 5%)	0.60 (0.34–1.06)	0.70 (0.39–1.27)	0.69 (0.38–1.25)	0.71 (0.39–1.30)

Odds Ratios (95% CI). *p < 0.05, **p < 0.01, ***p < 0.001.

M1 = Self-Rated Health, Cognition, Activities of Daily Living.

M2 = M1 + Age, Sex, Socio-economic Status.

M3 = M2 + Marital Status, Accommodation Type, Household Composition, Social Engagement.

M4 = M3 + Area Deprivation, Rurality, Centre.

Speech Therapist excluded due to low n.

To test the wave 1 findings, analysis investigated the association between individual allied health services and cognitive function in wave 2. The lower half of Table 4 shows the wave 2 logistic regression results. Average probabilities of using each service by cognitive function in wave 2 can be found in Supplementary Table S2. As evident in wave 1 data, there is a significant association between cognition and having a sight test and visiting the dentist in the year before interview across all models. In model 4, odds of a participant with cognitive impairment having their sight tested are 0.73 times smaller than odds for those without a cognitive impairment (p = 0.016), and odds of visiting a dentist 0.58 times smaller (p < 0.001).

In wave 1 there was evidence of an association between seeing the chiropodist and cognitive function in the base model, but this association did not reach significance in subsequent models. In wave 2 the reverse pattern is evident, with the base model not showing a significant association, but significance in models 2–4. In model 4, odds of a participant with cognitive impairment visiting a chiropodist in the four weeks prior to interview are 0.63 times smaller than odds for those without a cognitive impairment (p = 0.009). The base model showed a significant association between cognitive function and having a hearing test, with a person with a cognitive impairment having odds 1.39 times larger than those without a cognitive impairment, however this was attenuated when adjusting for age, sex, and socio-economic status in model 2, and did not reach significance in subsequent models. There are no significant associations between cognitive function and visiting the physiotherapist in wave 2, replicating the findings in wave 1.

For sight tests, dentist visits and chiropody (wave 2), people with a cognitive impairment had smaller odds of using the service compared to those without a cognitive impairment. To explore whether this could be related to people with cognitive impairment having greater difficulty accessing these services, a fifth model was estimated with wave 2 data, which included participant ratings of service accessibility using their usual forms of transport. The same exclusion criteria applied, with participants also excluded if they were missing data on accessibility of the service being investigated (analysis n = 1955 for sight tests; n = 1958 for the dentist; n = 1943 for the chiropodist).

The pattern of results with these slightly smaller samples matched that described above, with significant associations across models 1–4 for sight tests (Model 4 (M4) Odds Ratio (OR) = 0.72, 95% Confidence Intervals (CI) = 0.56–0.93, p = 0.013), and visiting the dentist (M4 OR = 0.57, 95% CI = 0.44–0.73, p < 0.001) and models 2–4 for visiting the chiropodist (M4 OR = 0.63, 95% CI = 0.45–0.89, p = 0.009). Adding accessibility (no difficulty/difficulty) to the model did not attenuate the association between cognitive function and sight tests (M5 OR = 0.72, 95% CI = 0.56–0.93, p = 0.012), visiting the dentist (M5 OR = 0.57, 95% CI = 0.44–0.73, p < 0.001), or chiropodist use (M5 OR = 0.63, 95% CI = 0.44–0.89, p = 0.008). These results indicate that accessibility in terms of travelling to a service does not account for the total differences in service use by cognitive impairment seen for sight tests, dentist or chiropodist visits.

## 4 Discussion

Overall, our results indicate that reported health service use is generally greater for those with worse health, according to two of the three indices used here. In terms of the Andersen model (Andersen, 1995), when predisposing factors (including age, gender, socio-economic status, deprivation indices) and enabling factors (including social support and engagement) are considered, the relationship between health need and service use remains. Analysis of service categories in wave 1 showed increases in use of medical services including hospital, GP, and nursing services as a function of SRH and activities of daily living, with service use increasing as health declined. Cognitive impairment, in contrast, was not associated with use of these medical services. It may be that functional impairment (reflected in IADL disability) triggers use of these services, rather than cognitive impairment per se. Another possibility is that our sample did not include people with severe cognitive impairments who could not participate fully in interviews and so were excluded from analysis. It may be that people with more severe cognitive impairment are more likely to use medical services than those with a relatively mild cognitive impairment. However, whilst having to exclude people with missing data is a limitation of the study, the data were sufficient to detect significant differences in social care and allied health service use by cognitive function. Social care service use increased with poorer health as indicated by cognitive function and activities of daily living but was not significantly associated with SRH when factoring in covariate and modifier variables. Probability estimates suggested that people with poor SRH have a lower probability of using social care than those with excellent SRH. Whilst not significant, this pattern could be an example of the inverse care law (Hart, 1971), with those most in need of care the least likely to receive it. Alternatively, each model holds the other health measures at a constant, in this case cognitive function and activities of daily living, and therefore it could suggest that when need (as indicated by ADL/IADL disability) is held constant, people who receive social care rate their health as better than those who do not receive such care.

There is some evidence of an association between allied health service use and SRH, with increased use for fair compared to excellent SRH, but decreased use for poor compared to fair SRH, suggesting a non-linear relationship between SRH and allied health service use. This could suggest that those in excellent SRH do not feel the need to use an allied health service in the same way as those with fair SRH. Those with poor SRH might be using more intensive services that take priority over allied health services, or that allied health services do not provide the support that they need. A key finding from the wave 1 analysis is that people with a cognitive impairment were at reduced odds of using an allied health service than those without a cognitive impairment. Follow-up analysis considering individual allied health services showed that the difference by cognitive impairment was evident for sight checks and dentist visits, a finding replicated with wave 2 data, which remained when adding a further enabling factor, service access (transportation), to the model. There was some evidence of differences in chiropodist use as a function of cognitive impairment, however this was inconsistent between waves 1 and 2, with further study needed to confirm the result.

It is noteworthy that of all the individual allied health services and service categories, sight tests and visiting the dentist are the only services used by the majority of our sample, with all other services having a greater number of non-users than service users. This suggests a general need for these services across the population, from which people with a cognitive impairment are excluded. The association of cognitive impairment and sight checks and dentist visits remains significant after factoring in access using usual transport, indicating transportation alone does not account for the difference. Allied health services are often paid-for services in the UK, not always publicly funded and free at the point of service, like services available through the National Health Service, although this would also apply to hearing checks where cognitive impairment did not reduce service use. There is evidence to show income-related inequality in dental service use of older adults (Listl, 2010), and that lower socio-economic positions are associated with poorer oral health (Tsakos, Demakakos, Breeze, & Watt, 2011). However, socio-economic status and area material deprivation were included from model 2 onwards and therefore held constant in the analysis, suggesting that this did not account for all differences in service uptake. One possibility is that current sight tests and dental examinations are not adapted to the cognitive needs of individuals with a cognitive impairment and are therefore not accessible for the individual. Alternatively, human capital models suggest that use of preventative services are influenced by considerations of investment in a future self (Carrieri & Bilger, 2013). There is evidence to suggest that people living with dementia tend to focus on the present rather than envisage the future (Heaton et al., 2020, pp. 1–21). Differences in the use of sight tests and visiting the dentist, services which offer preventative as well as therapeutic care, may reflect differences in future orientation and inclination to invest in the future self.

In general, our findings are consistent with the pattern of service use found by Burholt et al. (1997) 20 years ago, with people with a cognitive impairment generally more likely to use social care services, and less likely to use allied health services. Similarly, there were no significant differences found for GP use. However, in contrast to the significant difference found by Burholt et al. (1997) in their urban group, we did not find significant differences in use of nursing services between those with and without cognitive impairment. Many of the findings of Burholt et al. (1997) failed to reach significance, potentially due to the small sample size and lack of control for potential confounding variables. Similarly, the differing sample sizes across urban and rural groups may explain why the services reaching significance varies across these groups. An important feature of the current study is the inclusion of other health indicators, and potential covariate and modifier factors including demographic, social, and environment factors, that may affect the relationship between cognitive impairment and service use. The inclusion of these additional factors, the comparatively large sample size, and the inclusion of a wider range of allied health services, provides a more nuanced and robust analysis of the current relationship between health (including cognitive function) and service use.

### 4.1 Study strengths and limitations

A strength of this study is the large population-based sample, with participants randomly sampled from across North and South Wales, including people residing in the community and in care settings. The longitudinal aspect of the study allowed replication of findings across waves at two time-points, suggesting that the findings are stable over the two-years between interviews. As with all population-based longitudinal studies, there are missing data. Imputation of service use was not possible due to a lack of alternative indicators. Participants with missing data were therefore excluded from analysis, which is likely to include people with more severe cognitive impairment who may have struggled to answer questions. Similarly, it was not possible to interview people with severe dementia. People with more severe cognitive impairment are therefore likely to be underrepresented. As a population-based survey the sample reflects the makeup of the wider population of Wales, a limitation of this approach is there were insufficient numbers of people from ethnic minority backgrounds to include ethnicity in analyses.

A strength of this study is the wide range of health and social care services investigated. It was not possible to look at actual service use, with self-report used as a proxy indicator. Self-report relies on memory and is likely to be more difficult for people with cognitive impairment. It was also not possible to consider the quality or intensity of service use, or to look at the degree to which services were offered and accepted or rejected. Health was considered an indicator of need, with need inferred rather than measured. Whilst a limitation in some ways, taking a broad approach to health, and consequently need, allows examination beyond individual symptoms and health specific needs to see a bigger picture of health service inequalities. Finally, it is difficult to measure levels of social care in care homes, however, the pattern of findings for social care service use are largely in the direction expected, suggesting that inclusion of participants in care homes has not distorted results.

### 4.2 Policy implications

Eyesight checks play an important role in maintaining health. Whilst their primary purpose is to monitor changes in vision and detect eye conditions that could lead to sight loss (e.g. cataracts, glaucoma and age-related macular degeneration), they are also able to detect other health conditions such as diabetes, hypertension, and hypercholesterolemia (Schaneman, Kagey, Soltesz, & Stone, 2010). Furthermore, poor vision is a significant risk factor for falls and fractures in older adults (Lord, 2006), with 21% of total costs of treating accidental falls in the UK spent on those with visual impairment, and 10% of falls directly attributable to visual impairment (Scuffham, Legood, Wilson, & Kennedy-Martin, 2002). Visual impairment is also associated with increased risk of depression (Hayman et al., 2007). A review by Evans and Rowlands (2004) found that 20–50% of older people have undetected reduced vision, the majority of whom had correctable problems, highlighting the importance of sight checks for older adults.

Oral health can impact quality of life, with teeth affecting physical, psychological, and social aspects of a person's life (Masood, 2017). There is evidence of associations between oral health and other health conditions including respiratory infection (Scannapieco, 1999) and chronic obstructive pulmonary disease (Liu et al., 2012). Furthermore, there is some suggestion that people with Alzheimer's disease have “reduced ability to identify pain or discomfort associated with periodontitis, gingival bleeding, missing teeth, and decay and may not report relevant oral health complaints” (Ming, Hsu, Yen, & Lan, 2019, p. 173), making it even more important for this population to have regular dental checks to identify problems early on.

Despite changes to health and social care over the last 20 years and increased recognition of dementia and associated needs through national dementia plans and National Institute for Health and Clinical Excellence (NICE) guidelines (NICE, 2006), there remains differences in service use between those with and without cognitive impairment. Apparent inequalities in the use of sight checks and dentist visits as a function of cognitive impairment is indicative of a system where services continue to operate in relative isolation, focusing on individual conditions, with people still falling through gaps in services which are preventative as well as therapeutic. It is important not to overlook regular use of routine health check services when faced with other significant health challenges, and all services need to implement pathways, such as the UK Dementia Eye Care pathway (Hancock, Shah, Edgar, & Bowen, 2015) to facilitate use by vulnerable groups. As populations age and service providers and commissioners look to increase the sustainability of their services, uptake of health check and prevention services is a key area to target. Early identification of illnesses and consequently reduced need for intensive and costly care packages, such as long-term hospital stays, not only increases the likelihood of a better outcome for individuals but is also more cost-effective for service providers and the economy in the long-term (National Health Service Improvement, 2018). It is important to ensure that those experiencing health difficulties continue to receive health check and prevention services related to other conditions. As evident in the current study, those experiencing one health problem may lose out on care in other areas. Better integration of health and social care systems is one possible route to minimise disruption in routine care, which may get overlooked when focusing on other health problems. As described by the World Health Organisation “building an age-friendly world, requires a transformation of health systems away from disease-based curative models and towards the provision of integrated care that is centred on the needs of older people” (World Health Organisation, 2015 p. viii).

## 5 Conclusions

This paper examines the association between use of a variety of health and social care services and health in older adults in Wales, to understand current service use, and identify gaps in service uptake. As expected, medical and social care service use typically increased as health decreased. However, there was a clear gap in service uptake for allied health services, particularly sight checks and visiting the dentist, with those with a cognitive impairment at significantly lower odds of using these services than those without. With global concerns about the sustainability of health services in the context of ageing populations, these findings highlight a key area for service providers and commissioners to target to improve the well-being of those with cognitive impairment, potentially reducing demand on more intensive and costly care packages and increasing the sustainability of health care services overall.

## Declaration of competing interest

The authors declare that they have no known competing financial interests or personal relationships that could have appeared to influence the work reported in this paper.

## Author statement

Catherine MacLeod: Conceptualization; Investigation; Methodology; Formal analysis; Funding acquisition; Writing – original draft. Feifei Bu: Conceptualization; Methodology; Writing – review & editing. Alasdair Rutherford: Conceptualization; Methodology; Writing – review & editing. Judith Phillips: Conceptualization; Investigation; Methodology; Funding acquisition; Writing – review & editing. Robert Woods: Conceptualization; Investigation; Methodology; Funding acquisition; Supervision; Writing – review & editing.

## References

[bib1] Andersen R.M. (1995). Revisiting the behavioral model and access to medical care: Does it matter?. Journal of Health and Social Behavior.

[bib2] Bibby P., Brindley P. (2013). Urban and rural area definitions for policy purposes in England and Wales: Methodology (v1.0). https://assets.publishing.service.gov.uk/government/uploads/system/uploads/attachment_data/file/239477/RUC11methodologypaperaug_28_Aug.pdf.

[bib3] Burholt V., Wenger G.C., Scott A. (1997). Dementia, disability and contact with formal services: A comparison of dementia sufferers and non-sufferers in rural and urban settings. Health and Social Care in the Community.

[bib4] Canvin K., MacLeod C.A., Windle G., Sacker A. (2018). Seeking assistance in later life: How do older people evaluate their need for assistance?. Age and Ageing.

[bib5] Carrieri V., Bilger M. (2013). Preventative care: Underused even when free. Is there something else at work?. Applied Economics.

[bib6] Clare L., Wu Y.-T., Teale J.C., MacLeod C., Matthews F., Brayne C. (2017). Potentially modifiable lifestyle factors, cognitive reserve, and cognitive function in later life: A cross-sectional study. PLoS Medicine.

[bib7] Cognitive Function & Ageing Study. (n.d.) (2015). CFAS II additional data information. http://www.cfas.ac.uk/files/2015/07/CFAS-II-additional-data-information.pdf.

[bib8] Copeland J.R.M., Dewey M.E., Saunders P. (1991). The epidemiology of dementia: GMS-AGECAT studies of prevalence and incidence, including studies in progress. European Archives of Psychiatry and Clinical Neuroscience.

[bib9] Dixon-Woods M., Kirk D., Agarwal S., Annandale E., Arthur T., Harvey J., Sutton A. (2005). Vulnerable groups and access to health care: A critical interpretive review. http://www.netscc.ac.uk/hsdr/files/project/SDO_FR_08-1210-025_V01.pdf.

[bib10] Evans B.J.W., Rowlands G. (2004). Correctable visual impairment in older people: A major unmet need. Ophthalmic and Physiological Optics.

[bib11] Folstein M.F., Folstein S.E., McHugh P.R. (1975). “Mini-mental state”: A practical method for grading the cognitive state of patients for the clinician. Journal of Psychiatric Research.

[bib12] Hancock B., Shah R., Edgar D.F., Bowen M. (2015). A proposal for a UK dementia eye care pathway. Optometry in Practice.

[bib13] Hart J.T. (1971). The inverse care law. Lancet.

[bib14] Hayman K.J., Kerse N.M., La Grow S.J., Wouldes T., Robertson M.C., Campbell A.J. (2007). Depression in older people: Visual impairment and subjective ratings of health. Optometry and Vision Science.

[bib15] Heaton J., Martyr A., Nelis S.M., Marková I.S., Morris R.G., Roth I., Clare L. (2020). Future outlook of people living alone with early-stage dementia and their non-resident relatives and friends who support them.

[bib16] Katz S. (1983). Assessing self-maintenance: Activities of daily living, mobility, and instrumental activities of daily living. Journal of the American Geriatrics Society.

[bib17] Lawton M.P., Brody E.M. (1969). Assessment of older people: Self-maintaining and instrumental activities of daily living. The Gerontologist.

[bib18] Licchetta M., Stelmach M. (2016). Fiscal sustainability analytical paper: Fiscal sustainability and public spending on health. https://obr.uk/docs/dlm_uploads/Health-FSAP.pdf.

[bib19] Listl S. (2010). Income-related inequalities in dental service utilization by Europeans aged 50+. Journal of Dental Research.

[bib20] Liu Z., Zhang W., Zhang J., Zhou X., Zhang L., Song Y. (2012). Oral hygiene, periodontal health and chronic obstructive pulmonary disease exacerbations. Journal of Clinical Periodontology.

[bib21] Long J.S., Freese J. (2014). Regression models for categorical dependent variables using Stata.

[bib22] Lord S.R. (2006). Visual risk factors for falls in older people. Age and Ageing.

[bib23] Lubben J. (1988). Assessing social networks among elderly populations. Family & Community Health.

[bib24] Lubben J., Blozik E., Gillmann G., IIiffe S., von Renteln Kruse W., Beck J.C. (2006). Performance of an abbreviated version of the Lubben Social Network Scale among three European Community–dwelling older adult populations. The Gerontologist.

[bib25] Masood M., Newton T., Bakri N.H., Khalid T., Masood Y. (2017). The relationship between oral health and oral health related quality of life among elderly people in United Kingdom. Journal of Dentistry.

[bib26] Matthews F.E., Arthur A., Barnes L.E., Bond J., Jagger C., Robinson L., on behalf of the Medical Research Council Cognitive Function and Ageing Collaboration (2013). A two‐decade comparison of prevalence of dementia in individuals aged 65 years and older from three geographical areas of England: Results of the Cognitive Function and Ageing Study I and II. Lancet.

[bib27] Ming Y., Hsu S.-W., Yen Y.-Y., Lan S.-J. (2019). Association of oral health–related quality of life and alzheimer disease: A systematic review. The Journal of Prosthetic Dentistry.

[bib28] National Institute for Health and Clinical Excellence (2006). Dementia: Supporting people with dementia and their carers in health and social care (NICE guideline CG42). https://dementiapartnerships.com/wp-content/uploads/sites/2/nice-cg42.pdf.

[bib29] National Health Service Improvement (2018). Guide to reducing long hospital stays. https://improvement.nhs.uk/documents/2898/Guide_to_reducing_long_hospital_stays_FINAL_v2.pdf.

[bib30] Office for National Statistics (2016). The national Statistics socio-economic classification (NS-sec). https://www.ons.gov.uk/methodology/classificationsandstandards/otherclassifications/thenationalstatisticssocioeconomicclassificationnssecrebasedonsoc2010.

[bib31] Office for National Statistics (2019). Health state life expectancies, UK: 2016 to 2018. https://www.ons.gov.uk/peoplepopulationandcommunity/healthandsocialcare/healthandlifeexpectancies/bulletins/healthstatelifeexpectanciesuk/2016to2018/pdf.

[bib32] Prince M., Knapp M., Guerchet M., McCrone P., Prina M., Comas-Herrera M., Salimkumar D. (2014). Dementia UK: Update. https://www.alzheimers.org.uk/sites/default/files/migrate/downloads/dementia_uk_update.pdf.

[bib33] Røsvik J., Michelet M., Engedal K., Bergh S., Bieber A., Gonçalves-Pereira M. (2020). Development of best practice recommendations to enhance access to and use of formal community care services for people with dementia in europe: A delphi process conducted by the actifcare project. Aging & Mental Health.

[bib34] Sarkisian C.A., Hays R.D., Mangione C.M. (2002). Do older adults expect to age successfully? The association between expectations regarding aging and beliefs regarding healthcare seeking among older adults. Journal of the American Geriatrics Society.

[bib35] Scannapieco F.A. (1999). Role of oral bacteria in respiratory infection. Journal of Periodontology.

[bib36] Schaneman J., Kagey A., Soltesz S., Stone J. (2010). The role of comprehensive eye exams in the early detection of diabetes and other chronic diseases in an employed population. Population Health Management.

[bib37] Scuffham P.A., Legood R., Wilson E.C.F., Kennedy-Martin T. (2002). The incidence and cost of injurious falls associated with visual impairment in the UK. Visual Impairment Research.

[bib38] Tsakos G., Demakakos P., Breeze E., Watt R.G. (2011). Social gradients in oral health in older adults: Findings from the English longitudinal survey of aging. American Journal of Public Health.

[bib39] Walters K., Iliffe S., Orrell M. (2001). An exploration of help-seeking behaviour in older people with unmet needs. Family Practice.

[bib40] Welsh Government (2015). Welsh index of multiple deprivation: A guide to analysing indicator data. https://dera.ioe.ac.uk/28905/1/170413-wimd-indicator-data-guidance-en.pdf.

[bib41] Welsh Government (2019). Annual population survey: Ethnicity. StatsWales, v.June 2019. https://statswales.gov.wales/Catalogue/Equality-and-Diversity/Ethnicity/ethnicity-by-age.

[bib42] Woods R., Windle G., Burholt V., Brayne C., Bennett K., McCracken C., Macleod C. (2019). Cognitive function and ageing study - Wales: Waves 1-2, 2011-2016.

[bib43] World Health Organisation (2015). World report on ageing and health. http://www.who.int/iris/bitstream/10665/186463/1/9789240694811_eng.pdf.

