# Supplementary material for: Cognitive impairment negatively impacts allied health service uptake: Investigating the association between health and service use
Source: SSM Popul Health. 2020 Dec 13;13:100720. doi: 10.1016/j.ssmph.2020.100720 (PMC7750552; doi:10.1016/j.ssmph.2020.100720)
Supplement: Multimedia component 1 [file mmc1.pdf]

## Supplementary Materials

*Table S1. Average marginal effects of service use by health (n=3153)*

| | Model 1 | Model 2 | Model 3 | Model 4 |
|---|---|---|---|---|
| SOCIAL CARE | | | | |
| **Self-Rated Health** | | | | |
| Good vs Excellent | -0.015 | -0.010 | -0.013 | -0.012 |
| Fair vs Excellent | -0.015 | -0.006 | -0.010 | -0.008 |
| Poor vs Excellent | -0.034 | -0.015 | -0.018 | -0.016 |
| Fair vs Good | -0.000 | -0.004 | 0.003 | 0.003 |
| Poor vs Good | -0.019* | -0.005 | -0.006 | -0.004 |
| Poor vs Fair | -0.019* | -0.009 | -0.008 | -0.007 |
| **Cognitive Function** | | | | |
| Impairment vs No Impairment | 0.039*** | 0.029** | 0.025** | 0.025** |
| **Activities of Daily Living** | | | | |
| IADL vs No disability | 0.056*** | 0.043*** | 0.041*** | 0.042*** |
| ADL-IADL vs No disability | 0.168*** | 0.126*** | 0.127*** | 0.127*** |
| ADL-IADL vs IADL disability | 0.112*** | 0.083*** | 0.085*** | 0.085*** |
| HOSPITAL | | | | |
| **Self-Rated Health** | | | | |
| Good vs Excellent | 0.076** | 0.078*** | 0.078*** | 0.077*** |
| Fair vs Excellent | 0.170*** | 0.171*** | 0.172*** | 0.172*** |
| Poor vs Excellent | 0.229*** | 0.236*** | 0.237*** | 0.241*** |
| Fair vs Good | 0.094*** | 0.094*** | 0.094*** | 0.095*** |
| Poor vs Good | 0.153*** | 0.158*** | 0.159*** | 0.164*** |
| Poor vs Fair | 0.059 | 0.064 | 0.065 | 0.068 |
| **Cognitive Function** | | | | |
| Impairment vs No Impairment | -0.012 | -0.018 | -0.016 | -0.013 |
| **Activities of Daily Living** | | | | |
| IADL vs No disability | 0.136*** | 0.137*** | 0.136*** | 0.140*** |
| ADL-IADL vs No disability | 0.179*** | 0.166*** | 0.169*** | 0.174*** |
| ADL-IADL vs IADL disability | 0.043 | 0.030 | 0.033 | 0.034 |
| ALLIED HEALTH | | | | |
| **Self-Rated Health** | | | | |
| Good vs Excellent | 0.011 | 0.013 | 0.015 | 0.014 |
| Fair vs Excellent | 0.029 | 0.032 | 0.036* | 0.036* |
| Poor vs Excellent | -0.027 | -0.025 | -0.015 | -0.013 |
| Fair vs Good | 0.017 | 0.019 | 0.021 | 0.022 |
| Poor vs Good | -0.038 | -0.037 | -0.030 | -0.028 |
| Poor vs Fair | -0.055* | -0.057* | -0.051* | -0.049* |
| **Cognitive Function** | | | | |

| | M1 | M2 | M3 | M4 |
|---|---|---|---|---|
| Impairment vs No Impairment | -0.072*** | -0.060*** | -0.049** | -0.047** |
| **Activities of Daily Living** | | | | |
| IADL vs No disability | 0.010 | 0.013 | 0.015 | 0.017 |
| ADL-IADL vs No disability | -0.005 | -0.000 | 0.004 | 0.008 |
| ADL-IADL vs IADL disability | -0.014 | -0.012 | -0.011 | -0.009 |
| **GENERAL PRACTITIONER (DOCTOR)** | | | | |
| **Self-Rated Health** | | | | |
| Good vs Excellent | 0.078*** | 0.080*** | 0.082*** | 0.081*** |
| Fair vs Excellent | 0.172*** | 0.176*** | 0.179*** | 0.180*** |
| Poor vs Excellent | 0.262*** | 0.275*** | 0.280*** | 0.284*** |
| Fair vs Good | 0.093*** | 0.096*** | 0.097*** | 0.098*** |
| Poor vs Good | 0.184*** | 0.195*** | 0.199*** | 0.203*** |
| Poor vs Fair | 0.091* | 0.099* | 0.102** | 0.104** |
| **Cognitive Function** | | | | |
| Impairment vs No Impairment | -0.031 | -0.031 | -0.029 | -0.026 |
| **Activities of Daily Living** | | | | |
| IADL vs No disability | 0.058* | 0.054* | 0.055* | 0.058* |
| ADL-IADL vs No disability | 0.068* | 0.056 | 0.057 | 0.062* |
| ADL-IADL vs IADL disability | 0.010 | 0.003 | 0.002 | 0.004 |
| **NURSING SERVICES** | | | | |
| **Self-Rated Health** | | | | |
| Good vs Excellent | 0.011 | 0.012 | 0.015 | 0.013 |
| Fair vs Excellent | 0.044* | 0.045* | 0.050** | 0.051** |
| Poor vs Excellent | 0.073* | 0.068* | 0.080** | 0.096** |
| Fair vs Good | 0.033* | 0.033* | 0.035* | 0.038* |
| Poor vs Good | 0.062* | 0.057* | 0.066* | 0.084** |
| Poor vs Fair | 0.029 | 0.023 | 0.031 | 0.045 |
| **Cognitive Function** | | | | |
| Impairment vs No Impairment | -0.003 | 0.003 | 0.009 | 0.016 |
| **Activities of Daily Living** | | | | |
| IADL vs No disability | 0.028 | 0.034* | 0.035* | 0.045** |
| ADL-IADL vs No disability | 0.095*** | 0.104*** | 0.107*** | 0.119*** |
| ADL-IADL vs IADL disability | 0.067** | 0.069** | 0.072** | 0.073** |

*p<0.05, **p<0.01, ***p<0.001

M1 = Self-Rated Health, Cognition, Activities of Daily Living.

M2 = M1 + Age, Sex, Socio-economic Status.

M3 = M2 + Marital Status, Accommodation Type, Household Composition, Social Engagement.

M4 = M3 + Area Deprivation, Rurality, Centre.

*Table S2. Average marginal effects of allied health service use for those with a cognitive impairment compared to those with no cognitive impairment in wave 1 (n=3152) and wave 2 (n=1968).*

| | | Model 1 | Model 2 | Model 3 | Model 4 |
|---|---|---|---|---|---|
| **Wave 1** | **SIGHT TEST** | -0.045* | -0.058** | -0.054* | -0.056** |
| | **HEARING TEST** | 0.008 | -0.008 | -0.007 | -0.007 |
| | **DENTIST** | -0.145*** | -0.076*** | -0.061** | -0.055** |
| | **CHIROPODIST** | 0.037* | 0.005 | 0.008 | 0.007 |
| | **PHYSIOTHERAPIST** | -0.013 | -0.003 | -0.001 | 0.002 |
| **Wave 2** | **SIGHT TEST** | -0.054* | -0.067* | -0.065* | -0.065* |
| | **HEARING TEST** | 0.052* | 0.038 | 0.035 | 0.034 |
| | **DENTIST** | -0.168*** | -0.121*** | -0.110*** | -0.106*** |
| | **CHIROPODIST** | -0.022 | -0.043* | -0.045** | -0.046** |
| | **PHYSIOTHERAPIST** | -0.019* | -0.014 | -0.014 | -0.013 |

*p<0.05, **p<0.01, ***p<0.001.

M1 = Self-Rated Health, Cognition, Activities of Daily Living.

M2 = M1 + Age, Sex, Socio-economic Status.

M3 = M2 + Marital Status, Accommodation Type, Household Composition, Social Engagement.

M4 = M3 + Area Deprivation, Rurality, Centre.
